# Examining the Current State of System Testing Methodologies in Quality Assurance

**DOI:** 10.1007/978-3-030-49392-9_16

**Published:** 2020-05-06

**Authors:** Rafaela Sophocleous, Georgia M. Kapitsaki

**Affiliations:** 6 University of Oslo, Oslo, Norway; 7 Monash University, Clayton, VIC Australia; 8 IT University of Copenhagen, Copenhagen, Denmark; 9 University of British Columbia, Vancouver, BC Canada; Department of Computer Science, University of Cyprus, Nicosia, Cyprus

**Keywords:** Software testing, Agile development, Regression testing, Smoke testing

## Abstract

Testing is an important phase of every software system, as it can reveal defects early and contribute to achieving high software quality. In this process of quality assurance, organizations are usually relying on one testing technique. However, a combination of techniques may prove more beneficial to the organization, as it might give the chance to discover a larger number of defects early. In order to examine the above, in the current work we present a survey on the use of system testing methodologies. We have gathered data from 252 individuals that reveal current trends in testing, such as whether requirements are used in the test case definition and whether the testing techniques used are affected by parameters, such as years of experience, whereas we examine the combination of smoke testing and regression testing. We also demonstrate an industrial use case, where this combination was applied, reducing the number of defects identified by the customer.

## Introduction

In Software Engineering, the testing phase can be defined as the process of validation and verification that a system meets the business and technical requirements of the customer and operates as expected. Through the phase of testing organizations are also trying to find defects with the purpose of resolving them and improving the system quality [5]. Developing a completely “error-free” software is almost impossible, but it is possible to produce very high quality software. Testing is moreover, the ability to test a specific process of the system and get every time the same result, verifying that the system behaves the same way.

Previous works surveyed testing techniques. Most approaches discuss the separation of the testing methodologies in different categories, such as dynamic and static testing, or focus on the best testing methodologies for specific programming languages [1]. Surveys that investigate the use of testing techniques in the software industry are also available, with one survey performed annually [6]. In this work, we examine how professionals are using system testing methodologies for Quality Assurance (QA) focusing on some of the choices they make (e.g. how defects are categorized), in an attempt to investigate how the combination of testing methodologies but also some choices performed during testing and the whole software engineering process can affect the testing success. Based on the results of the survey, we have applied the combination of testing methodologies in an industrial use case, specifically smoke testing and regression testing, to verify its usefulness. The main contribution of our work in relation to previous works are that: 1) we study the current state of adoption of basic testing techniques for QA, and 2) we have used a practical case to demonstrate that the combination of more than one testing techniques can decrease defect detection by the customer. Most of the participants of our study employ Agile methodologies, so our results are valid for Agile practices.

The rest of the paper is structured as follows. Section 2 presents related work in the area. Section 3 shows the process that we have followed in order to create the questionnaire and the data collection process. Section 4 presents the main results of the survey, whereas the results of the industrial use case and limitations of the study are also presented. Finally, Sect. 5 concludes the paper.

## Related Work

Previous works present the available testing methods and tools, such as techniques for software functional testing [3] or regression testing [7]. Other works are dedicated to specific programming languages, such as techniques for dynamic program analysis and test generation for JavaScript [1]. Previous surveys have also addressed testing techniques in the industry. The State of Testing 2019 Annual Report gathered approximately 1,000 participants from more than 80 countries and studied various aspects of testing [6]. This study contains some similar questions with our survey, such as the size of the testing team, and the development lifecycle model used but focuses overall on more generic aspects, such as the tester’s education, techniques and methodologies used and the tester’s personal development. Its results are very useful and informative but have a different orientation from our work that aims mainly on identifying the usefulness of the combination of some testing techniques in quality assurance teams.

A survey of practices in software testing methods and tools (STMTs) focusing on capabilities, limitations, improvements and needs of the tools is presented in [4]. The main conclusions were that tool usage by the organizations was considerably lower than method usage, that there is limited tool support for testing methods and that there is a high demand for interoperability between methods and tools. In contrast to our work, this work focused on how well the software testing process and activities are supported by the existing methods and tools.

In relation to previous work, we present the current state of testing techniques and, although we have a smaller number of participants than the very useful State of Testing 2019 Annual Report, we also have a different focus with the aim of understanding test cases, connect testing techniques with other factors and identify combinations of testing techniques for the quality assurance team that assist in producing a lower number of defects. In addition, we have used an industrial use case, in order to examine whether combinations can provide better results in terms of defect detection before the system proceeds to production.

## Study Design

The main aim of the current study is to understand the adoption of testing techniques with a focus on combining methodologies. Based on this aim, the survey questions were created in order to give emphasis on this combination and allow participants to provide information on their testing techniques and results. The main Research Questions (RQs) that our survey intended to answer are the following:

- **RQ1.** How are the test cases created, especially with relation to the requirements? How many defect categories does an organization use?
- **RQ2.** Which testing techniques do organizations employ? Are there any factors that affect the techniques used, such as the years of experience of the practitioner or the software lifecycle model used?
- **RQ3.** Are combinations of smoke and regression testing before the system goes to production usual? Do they provide better results in terms of defects detected by the customer?

The questionnaire^1^ created for the purpose of the survey consists of 28 questions. The first part covers demographic data (e.g. country, age, years of experience), whereas the second part is dedicated to how testing is used within the organization, referring to generic techniques, their combination, the lifecycle model used and specific tools and environments adopted for testing purposes, building on the above research questions. The survey participants were informed about the purpose of the study and they had to agree to the consent form, in order to be able to proceed with answering the questionnaire. In order to reach a large number of participants, the survey questionnaire was distributed to local companies and was posted on social media in groups that are relevant to testing and testing practices, in an attempt to gather participation from different countries. Individual interviews with leaders of quality assurance teams from Cyprus (15 practitioners) were also performed and the main conclusions are included in the paper. However, no formal interview process was followed.

## Results and Discussion

### Demographics

**Fig. 1.**
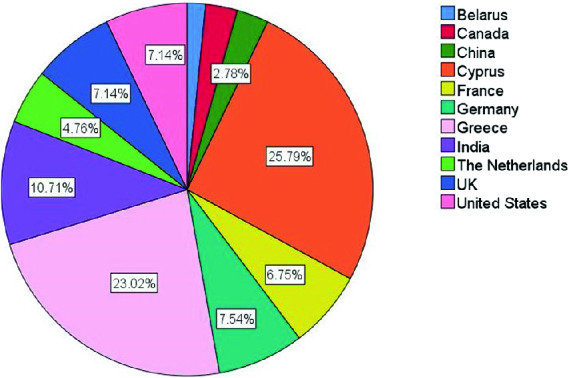
Participation per country.

252 responses were gathered, 56% of our respondents are male, 43.7% female, whereas 4% (1 participant) chose not to provide this information. Individuals of different age were reached: 23.4% are between 18 and 30, 51.2% between 31 and 40, 23.8% between 41 and 60, and 1.6% above 60 years old. The individuals have various roles in the organization, including automation testers, test engineers, software engineers, performance testers, QA director and test leads, with most participants having more than one role. Most have a role in testing (90.9%), whereas the rest are developers, analysts or managers. Individuals from 11 countries took part in the study, although a large percentage of the participants are from Cyprus (25.79%), where the survey was designed, whereas many participants from Greece were also reached due to the proximity of the countries (23.02%). Figure 1 shows the participation per country.

### RQ1. Test Cases and Defects Categorization

**Test Cases and Requirements.** We asked the participants how much (%) of the requirements are covered by the test cases. Many companies are not using all system requirements, in order to design their test cases: 10.3% of the participants are not using the requirements at all, 29% are using 30% of the requirements, 8.3% are using them by 40%, the majority are using 50% of the requirements (39.7%), only 4.8% are using all requirements, and the remaining (almost 8%) are using 60–80% of them. This causes several problems because it means that the system is not tested properly in order to detect all errors before its delivery to the customer, as creating test cases from requirements to test the behavior of a software system is a main aspect of black-box testing [8].

Through the interviews with the leaders of QA teams of different companies about the results that we received for the specific question, participants commented that the team members of the QA team are not well trained to know some basic guidelines on how to write a test case or sometimes they do not have much time to execute these test cases. It has also been reported by most that they only use positive paths, in order to design their test cases. Through the interviews, it was identified that very few testing techniques are known or are used by the leaders of quality assurance teams in order to write their test cases: functional, requirement, positive, stress and exploratory. However, this raises several problems regarding the software quality assurance team and ultimately the quality of the system that is delivered to the client, because negative testing is not used. It is important that a system is tested for both positive and negative paths to make sure that everything works as agreed with the client [2].

**Categories of Identified Defects.** We asked participants to indicate the defects categories they are using. We included indicative categories but allowed participants to mention more categories. The answers are shown in Table 1. Most participants (25.9%) are using the following 3 levels: Critical, Highest/High and Medium, with 53.6% using three levels overall but not the same levels in all cases. 39.7% are using two categories, 2.8% are using only one and 3.6% are using 4 or 5 different categories (1 participant did not provide any answer). Based on the interviews conducted, most of the companies tend not to pay enough attention to understand why there are defects that are categorized into the above specific categories, when the system is delivered to the customer. Also, most of the leaders of the quality assurance teams have been told that they simply collect the defects that can be identified by the client but they do not take into account the defects that are raised by the quality assurance team.

### RQ2. Testing Techniques and Relevant Factors

**Fig. 2.**
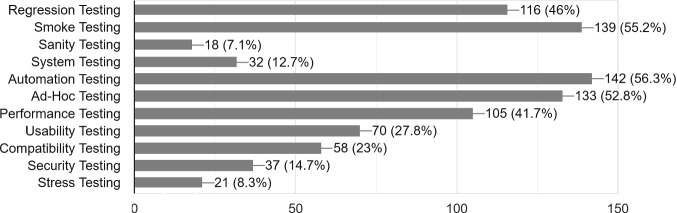
Testing techniques used.

Various testing methodologies are employed by the participants. Most are employing black box testing (87.3%), whereas some are using both black and white box (11.1%) and only 3 participants (1.2%) are only using white box testing. This is an expected result, as our survey targeted members of the QA team and the results we report concern primarily black box testing. Almost all participants are using both static and dynamic testing (96.8%) and almost all are relying both on functional and non-functional testing (94%). Figure 2 lists various specific techniques used by participants. Unit testing was also mentioned by a small number of participants but it is not considered, as we assume that it applies to almost all involved organizations, if we consider their development teams. 2 participants are using only one technique, most (35.7%) are using 3 different techniques, followed by 5 techniques in 21% of the participants, 4 techniques in 18.7% and 2 techniques in 14.7%. A small percentage (2.4%) is employing 8 or more techniques. Although automation testing appears in 56.3% of cases, in a more general question on automation, the use of some sort of automation was reported by 77% of the participants.

**Table 1.** Categories used to characterize defects.

Categories	Use percentage	Categories	Use percentage
Critical, Highest/High, Medium	25.9%	Highest/High, Medium, Low	3.2%
Critical, Highest	23.9%	Critical/Highest/High	2%
Critical/Highest, High	15.9%	Critical, High, Medium, Low	1.6%
Critical/Highest/High, Medium	14.3%	Critical, Highest, High, Medium, Low	1.6%
Highest, High, Medium	5.6%	Medium	0.8%
Critical, Highest, High	4.8%	Critical, High, Medium, Low, Block	0.4%

We examined whether the testing techniques employed are affected by the years of experience of the engineer. We run one-way ANOVA and observed a statistically significant difference in the use of regression testing (*p* = 0.000), smoke testing (*p* = 0.000), system testing (*p* = 0.000), automation testing (*p* = 0.001), performance testing (*p* = 0.009), and usability testing (*p* = 0.000). From Table 2 we observe that there is a decrease in the use of system testing and performance testing by individuals with larger experience and also a slight decrease in the use of usability testing, whereas automation testing is used widely in all groups.

**Table 2.** Testing techniques with significant differences per years of experience.

Experience (years)	Regression testing	Smoke testing	System testing	Automation testing	Performance testing	Usability testing
1–3	47.5%	67.5%	20%	52.5%	42.5%	60%
3–5	51.7%	41.4%	37.9%	55.2%	65.5%	27.6%
5–10	26.8%	70.1%	2.4%	48%	35.4%	20.5%
>10	86.3%	17.6%	11.8%	82.4%	33.3%	25.5%

We examined whether the adopted software development lifecycle model affects the testing techniques used, as for instance, automation testing is used mainly in Agile methodologies. 61.9% of the participants are using Agile or iterative incremental models (59.9% are using Agile methods). The next most frequent practice is DevOps (35.7%), followed by the waterfall model (2%), whereas V-shaped model was mentioned by 1 participant. We observed a statistically significant difference in the use of many testing techniques: sanity testing (*p* = 0.000), system testing (*p* = 0.000), automation testing (*p* = 0.000), performance testing (*p* = 0.000), usability testing (*p* = 0.000), compatibility testing (*p* = 0.000), security testing (*p* = 0.000) and stress testing (*p* = 0.032). In our dataset we observed that automation testing is used equally in Agile and waterfall methods (40.4% and 40% respectively), but it is used more in DevOps (85.6%). Sanity and system testing are far more common in the waterfall model (60% and 80% respectively), whereas they are used only in small percentages in Agile and DevOps methods. Performance and usability testing are used more in Agile than in other techniques. Security testing is overall less common but more frequent in Agile (20%) than in other methodologies. Compatibility testing is used in Agile and waterfall methods (31.1% and 40% respectively), but is less common in DevOps (6.7%). Finally, stress testing is used overall less but is more common in the waterfall model (20%) and rare in DevOps (1.1%).

### RQ3. Combination of Smoke and Regression Testing Before Production

Most participants (95.6%) responded that the defects that have been raised by the quality assurance team or client will be reduced if any combination of testing techniques will be applied to the system. We then provided the following specific choices of testing techniques asking the participants to provide their own additional combination based on the available testing techniques used also in other survey questions (e.g. sanity testing): 1) smoke testing and regression testing, 2) integration testing, 3) all the above. Smoke testing is generally a surface level testing to ensure that the build the development team has provided to the QA team can be accepted for further testing, whereas regression testing is testing on a deeper level. In integration testing, individual units are combined and tested together as a group. Most of the participants (91.67%) believe that the most effective combination of testing techniques before the system will be delivered to the production is the smoke and regression testing. Only 1.98% gave a positive answer for integration testing, and 6.35% would prefer a combination of all (smoke testing, regression testing and integration testing), with no participant providing any other specific combination. However, the majority of participants is currently not using this combination: 89.3% are not using smoke testing along with regression testing before production, 9.9% do and the remaining 0.8% were not sure or do not know.

### Industrial Use Case

The combination of smoke and regression testing techniques was employed in the framework of an industrial use case in the local industry in Cyprus for a total duration of two months and a half. The use case concerns the testing of a web application in the healthcare domain. The organization uses development sprints and was initially employing only smoke testing. We are not providing however, more information on the organization and the specific system, as the organization asked for the anonymous use of the data. In order to examine the combination of techniques, both smoke and regression testing were used for 5 consecutive sprints. We run a t-test to examine whether there is any difference in the number of defects detected, when the system was delivered to the customer, before and after the use of the combined testing. In the total of 31 sprints, we observed that the difference in the results is statistically significant (*p* = 0.000) with considerably less defects being detected when the combination of testing techniques is used (Table 3). In order to examine whether the number of changes coming from development affects the number of defects detected (since some sprints contain more changes than others), we used Pearson correlation, but no correlation was detected (*r* = 0.052, *p* = 0.782), indicating that this is not a parameter that affects our results. Thus, this combination of testing techniques is very effective for the QA team, in order to identify the defects before the system will be deployed on production environment, that is an expected outcome as testing is strengthened.

**Table 3.** Difference in the number of defects in the industrial use case.

Testing technique	N	Mean	Std. dev.
Smoke testing only	16	263.81	90.385
Smoke and regression testing	5	55.2	13.989

### Limitations

Our study is affected by *external validity*, referring to the extend we can generalize our findings. Our dataset is limited to 252 participants and expresses their views, whereas participants come from a limited number of countries (11 countries). Analyzing the state of testing techniques in other countries or populations may provide different results. *Construct validity*, i.e. the degree to which a test measures what it claims to be measuring, may have been affected by having specific views represented more in the data collected. More than one individuals from the same organization may have participated in the survey, affecting thus the overall results as they might contain in a larger degree the views of specific organizations. Although different testing techniques were mentioned in the questionnaire and were indicated by the participants (e.g. performance testing), we focused on the combination of specific testing techniques (i.e. smoke, regression and integration testing). Participants did not mention other combinations that could decrease the detection of defects, but mentioning specific techniques may have influenced their answer, having thus a negative effect on *conclusion validity* (the degree to which conclusions about the relationship among variables based on the data are correct). Finally, our study is not affected by *internal validity*.

## Conclusions

In this paper, we have presented the main results on a survey on system testing methodologies that focused on identifying which testing techniques are used, whether they are affected by the experience of the participants or by the lifecycle model used, as well as observing other common testing parameters, such as whether requirements are used for the creation of the test cases and the levels used to categorize defects. We have applied some results of the survey that refer to the combination of smoke and regression testing by the QA team in the framework of an industrial use case. The main conclusions drawn can be summarized to the following:

- Not many organizations are using requirements to create their test cases. 39.7% of participants are using half of the requirements, but as revealed also via the interviews the test cases are restricted to testing positive paths that may not provide the optimal results, as negative paths are neglected.
- Most organizations are using three levels to categorize defects but there is still a large number of organizations not giving appropriate importance to using more than one levels for the defects.
- In terms of testing techniques, various are being used in different phases of the development process. Ad-hoc testing is used by the majority of participants (52.8%), although it is usually not the only technique used. Some important types of testing, such as security testing and stress testing, appear to be less common (used by 14.7% and 8.3% of participants respectively).
- The choice of some testing techniques is affected by the years of experience of the expert or by the lifecycle model used, but since the participants number in each category is relatively small the observations drawn about these factors cannot be regarded conclusive.
- The combined use of smoke and regression testing can have a positive influence on the decrease of the number of defects detected by the customer and one main take-away message for organizations is to introduce this combination in their testing process by the QA team.

## Footnotes

1 https://forms.gle/4jprqc2t2f4yq9fg8.
